# Loneliness, Genetic Susceptibility, Brain Structure, and the Risk of Schizophrenia: A Large Prospective Study

**DOI:** 10.1093/schbul/sbaf179

**Published:** 2025-10-16

**Authors:** Jianing Wang, Zhongpeng Dai

**Affiliations:** The School of Public Health and Preventive Medicine, Monash University, Clayton VIC, Australia; State Key Laboratory of Brain and Cognitive Sciences, Laboratory of Neuropsychology & Human Neuroscience, The University of Hong Kong, Hong Kong 999077, China

**Keywords:** schizophrenia, loneliness, brain structure, genetic susceptibility

## Abstract

**Background and Hypothesis:**

Loneliness has been linked to adverse mental health outcomes, including psychosis, but its independent role in schizophrenia (SZ) development is unclear. We hypothesize that loneliness independently increases the risk of incident SZ and is associated with structural brain changes, especially in individuals with high genetic predisposition.

**Study Design:**

This prospective cohort study analyzed data from 491 613 UK Biobank participants aged 38-73 years without baseline SZ. Participants reported loneliness, provided demographic, socioeconomic, lifestyle information, and polygenic risk scores (PRSs) for SZ. Follow-up lasted until 2022, tracking incident SZ cases. Neuroimaging analyses examined gray matter volume associations with loneliness, considering genetic risk.

**Study Results:**

Over an average follow-up of 13.3 years, 675 individuals developed SZ. Those reporting loneliness (*n* = 91 017; 18.5%) had a significantly increased SZ risk, with an unadjusted hazard ratio (HR) of 3.84 (95% CI, 3.30-4.46). After adjusting for age, sex, socioeconomic factors, lifestyle, depression and anxiety symptoms, and PRS, the association remained stable (HR = 1.84; 95% CI, 1.36-2.49). Neuroimaging revealed widespread gray matter volume reductions linked to loneliness, notably in regions involved in emotion regulation and social cognition, such as the middle frontal gyrus, insula, hippocampus, and thalamus. These effects were more pronounced in individuals with higher SZ-PRS, with significant regional brain volume reductions among those with elevated genetic risk.

**Conclusions:**

Loneliness independently predicts increased SZ risk and is associated with widespread brain volume changes, especially in genetically vulnerable individuals. Addressing loneliness may be a key strategy to mitigate SZ development and brain structural alterations.

## Introduction

Schizophrenia (SZ) is a complex and debilitating neuropsychiatric disorder characterized by a diverse array of clinical symptoms, including hallucinations, delusions, cognitive deficits, and social dysfunction.^1^ Despite extensive research, the precise etiology and pathophysiological mechanisms underlying SZ remain incompletely understood.^2^ It suggests that the disorder results from an interplay of genetic, neurobiological, and environmental factors, which collectively influence its onset and progression.^3^^,^^4^

Among environmental influences, social factors, particularly loneliness, have received increasing attention for their potential role in the development and exacerbation of SZ. Loneliness is a prevalent and distressing experience among individuals with SZ, often linked to poorer clinical and social outcomes.^5^^,^^6^ It may not only be a consequence of social withdrawal but could also serve as a contributing factor that exacerbates symptoms and hinders recovery.^7^^,^^8^ Additionally, cognitive and social mechanisms, such as social cognitive biases, have been implicated in maintaining loneliness, indicating possible targets for intervention.^9^ Concurrently, genetic predisposition plays a crucial role in SZ, with polygenic risk scores (PRSs) emerging as valuable tools for quantifying genetic liability. While PRS has shown associations with symptom severity, neuroanatomical variability, and developmental exposures, its interpretation in relation to social risk factors like loneliness remains largely explored.^10-12^ Given these conditions, integrating PRS in SZ research is essential for examining the role of loneliness and its link to neurobiological pathology, facilitating early identification and personalized interventions.^13^^,^^14^

Neuroimaging studies have consistently revealed widespread structural brain abnormalities in SZ, including gray and white matter deficits, cortical thinning, and disrupted brain network organization. Longitudinal investigations suggest that these alterations are dynamic, evolving over the course of the illness and influenced by clinical factors such as relapse duration and treatment.^15^^,^^16^ Advanced imaging analyses have mapped specific regional changes associated with symptoms, such as auditory hallucinations, and elucidated large-scale network disruptions, including abnormalities in hub regions critical for global brain communication.^17-19^ Importantly, neurobiological correlates of loneliness—including regional brain volume reductions—have also been identified, supporting the notion that loneliness can influence brain structure in individuals with SZ.^20^

While considerable progress has been made in understanding the neurobiological underpinnings of SZ, the interplay between social experiences such as loneliness, genetic risk factors, and structural brain changes remains incompletely characterized. Integrating these domains could reveal critical insights into disease mechanisms and identify novel targets for intervention. This study is the first to examine the independent and interactive effects of loneliness, genetic predisposition, and brain structural alterations on SZ risk, utilizing a large prospective cohort along with PRS and neuroimaging data. By leveraging large-scale population data, our findings are expected to advance the understanding of the neurobiological and psychosocial mechanisms underlying SZ and contribute to more targeted prevention and treatment strategies.

## Methods

### Study Design and Population

The UK Biobank recruited more than 500 000 individuals aged 37 to 73 from across the United Kingdom between 2006 and 2010.^21^ This ongoing, population-based cohort study enrolls participants who are registered with the UK National Health Service (NHS). All participants provided written informed consent, and the study received approval from the North West Multicenter Research Ethics Committee. The present analysis was performed using UK Biobank data under application number 713374.

### Measures and Outcome Ascertainment

#### Loneliness

In the UK Biobank cohort, loneliness was assessed using two questions delivered through a touchscreen questionnaire. The initial question, “Do you often feel lonely?” was scored as 1 point for “yes” and 0 points for “no.” Participants were subsequently grouped into two categories: those with a score of 1 were identified as experiencing loneliness, whereas those with a score of 0 were classified as not lonely.

#### Outcome Definition

Schizophrenia diagnoses at baseline and during follow-up were verified using multiple data sources, including hospital admission records, primary care data, death registries, and self-reported physician diagnoses, which were further confirmed by nursing staff.^22^ The diagnosis was identified based on the International Classification of Diseases, Tenth Revision (ICD-10) code F20. Hospital admission information, routinely collected by the NHS, was linked to UK Biobank participants through anonymized numerical identification numbers. Death dates and causes were obtained from death certificates maintained by the NHS Information Center. Participants were followed from the time of enrollment until the first occurrence of SZ, death, or the cutoff date of October 30, 2022, whichever came first.

#### Genetic Data and PRS

The PRS for SZ was derived from the established PRS dataset available in the UK Biobank PRS Release. The detailed methods for calculating the PRS have been described in previous publications.^23^^,^^24^ Specifically, the UK Biobank used a standardized testing subset and predefined criteria for classifying diseases and traits. To develop the PRS algorithm, Bayesian techniques were employed, effectively integrating genetic data from diverse ancestral backgrounds and relevant phenotypic traits. Each single nucleotide polymorphism (SNP) included in the score was assigned a weight corresponding to its estimated effect size, as derived from existing genome-wide association studies. The individual risk score was calculated by summing the products of these weights and the number of risk alleles present for each SNP, with the number of risk alleles categorized as 0, 1, or 2. The total number of SNPs incorporated into the score varied based on the selection criteria, providing a comprehensive measure of genetic susceptibility across multiple genetic variants. Participants were then grouped into three categories based on their PRS distribution: low (lowest tertile), middle (middle tertile), and high (highest tertile).

#### Brain Imaging

From the original cohort of 501 968 UK Biobank participants, 63 573 individuals underwent brain MRI scanning, with each participant receiving a single scan at designated assessment centers. Imaging was conducted using a standardized Siemens Skyra 3T scanner equipped with a 32-channel head coil. The acquisition protocols and sequence parameters have been publicly available and detailed in previous publication.^24^ All MRI data were processed to derive image-based phenotypes (IDPs) within the UK Biobank dataset. The T1-weighted images, which offer high-resolution structural views of brain anatomy, were processed using pipelines that incorporated FMRIB Software Library (FSL) and FreeSurfer tools. Total gray matter volume (GMV) and total white matter volume (WMV) were calculated by adjusting the raw volumes with the head size scaling factor provided by UK Biobank to ensure normalization for head size. Cortical surfaces were reconstructed using FreeSurfer, and 34 IDPs related to regional volumes were extracted based on surface atlases. Volumes of subcortical structures were obtained using FSL’s FIRST tool, which also generated relevant 8 IDPs (a detailed list of descriptive statistics for brain regions were available in Supplementary Table S1). The FreeSurfer Desikan atlas was employed to delineate and quantify cortical and subcortical regions. To facilitate comparison and interpretation, all continuous measures of brain structure volumes were standardized using *Z* scores.

#### Covariates

In our analysis, we considered a variety of potential confounding variables based on prior research, including age, gender, ethnicity, pre-tax household income, educational level, body mass index, residential location, alcohol consumption, and smoking status. Sociodemographic information was collected at baseline. Ethnicity was categorized into six groups within the UK Biobank: white, multiracial, Asian, Black, Chinese, and other ethnicities. Educational attainment was simplified into two categories: individuals with or without a degree or professional qualification. Household income before tax was divided into two groups: <£31 000 or £31 000 and above. Residential setting was classified based on population density, with participants identified as living in either urban or rural areas. Body mass index was calculated from weight and height measurements taken by trained nurses. Smoking status was self-reported and categorized as never smoked, former smoker, or current smoker. Similarly, alcohol consumption history was self-reported and classified as never drank, former drinker, or current drinker. Overall health status was evaluated using a single-item rating, where higher scores indicate poorer health. Symptoms for depression and anxiety were assessed with a four-item Patient Health Questionnaire (PHQ-4), which measures participants’ depressive (such as feeling down or losing interest) and anxiety (such as feeling nervous or unable to control worrying).^25^ Higher total scores indicate more severe symptoms.

### Statistical Analysis

Baseline characteristics were summarized using means and standard deviations (s.d.) for continuous variables, and frequencies with corresponding percentages for categorical variables. To handle missing data in covariates, the fully conditional specification method through multiple imputation was applied. Differences in baseline features between individuals with and without SZ were assessed using Student’s *t*-test, Mann–Whitney *U*-test, or chi-squared tests, as appropriate.

To investigate the relationship between loneliness and the risk of developing SZ, a Cox proportional hazards model was employed. This model analyzed the duration from the initial loneliness assessment to the first diagnosis of SZ, death, or the censoring date of October 30, 2022. The Cox model is appropriate for time-to-event data, allowing us to assess the impact of loneliness on the timing of SZ onset while accounting for censoring and time-varying effects. To verify the proportional hazards assumption, we conducted the Schoenfeld residuals test, which indicated that the assumption was satisfied for all covariates. Initial analyses included an unadjusted model examining the crude association between loneliness and SZ (model 1). Subsequent models adjusted for potential confounders: model 2 included only sex and age; model 3 added education level and pre-tax household income; model 4 further incorporated BMI, alcohol consumption, and smoking status; model 5 included PRS; model 6 added the overall health rating; model 7 incorporated symptoms for depression and anxiety; and model 8 was fully adjusted, including all covariates.

Additionally, logistic regression analyses were conducted to explore associations between loneliness and various brain structure phenotypes, such as GMV, WMV, and regional cortical and subcortical volumes. Logistic regression is suitable for this cross-sectional analysis, allowing us to examine the relationship between loneliness and brain volume at a specific point in time. Prior to analysis, each phenotype was standardized to facilitate comparison across measures. Stratified analyses based on genetic risk categories (low, middle, high) were also performed within this context. To account for multiple comparisons in the brain phenotypes, we applied the Benjamini–Hochberg procedure for False Discovery Rate (FDR) correction.

**Table 1:** Descriptive Statistics for the Overall Sample and by Baseline Loneliness and Incident Schizophrenia

Characteristics	Participants^a^				
	Total (*N* = 491 163)	Loneliness at baseline		Incident schizophrenia	
		No (*n* = 400 541)	Yes (*n* = 91 072)	No (*n* = 490 938)	Yes (*n* = 675)
Follow-up, mean (SD), years	13.30 (2.00)	13.32 (1.97)	13.21 (2.15)	13.31 (1.99)	7.29 (3.96)
Reported loneliness	91 072 (18.5)	0	91 072 (100)	90 759 (18.5)	313 (46.4)
Gender					
Female	267 456 (54.4)	210 576 (52.6)	56 880 (62.5)	267 172 (54.4)	284 (42.1)
Male	224 157 (45.6)	189 965 (47.4)	34 192 (37.5)	223 766 (45.6)	391 (57.9)
Age, mean (SD), years	56.55 (8.09)	56.78 (8.08)	55.56 (8.05)	56.56 (8.09)	55.6 (8.43)

Abbreviation: SD, standard deviation.

a Data are presented as number (percentage) of participants unless otherwise indicated.

## Results

As shown in Table 1, an initial total of 501 968 individuals were recruited for the study. Exclusions included 907 participants who lacked loneliness assessment data, 497 individuals with a baseline diagnosis of SZ, 1428 respondents who selected “prefer not to answer,” and 7523 individuals who chose “do not know,” resulting in a final sample size of 491 613 participants, with 62 617 included in the subsequent brain imaging analyses. At baseline, participants’ ages ranged from 38 to 73 years, with a mean age of 56.55 years (SD = 8.09). The cohort consisted of 54.4% females and 45.6% males. Compared to those reporting no loneliness (*n* = 400 541; 81.5%), individuals who reported loneliness (*n* = 91 072; 18.5%) were more likely to be female, have lower educational attainment and income, higher BMI, PHQ-4 scores, and a history of smoking.

Over the ~16.63-year follow-up period (mean = 13.30 years, SD = 2.00; totaling 6 540 637 person-years), 675 participants developed SZ. Among these incident cases, 362 were from the non-lonely group, and 313 were from the lonely group. Participants, who developed SZ were more likely to be male, have lower income, higher BMI, a history of smoking, higher SZ PRS, and PHQ-4 scores. The detailed statistics for the comprehensive covariates were illustrated in Supplementary Table S2.

As shown in Figure 1, the unadjusted analysis indicated that individuals reporting loneliness had a significantly higher risk of developing SZ, with a hazard ratio (HR) of 3.84 (95% confidence interval [CI], 3.30-4.46). In model 2, adjusting for age and sex yielded an HR of 4.07 (95% CI, 3.49-4.74). Incorporating socioeconomic factors such as household income and education in model 3 resulted in an HR of 2.98 (95% CI, 2.42-3.65), maintaining statistical significance. Further adjustment for lifestyle variables, including BMI, smoking, and alcohol consumption in model 4, produced an HR of 3.62 (95% CI, 3.10-4.24). In model 5, after controlling for PRS, the HR was 3.80 (95% CI, 3.25-4.46), demonstrating that loneliness contributes to SZ risk beyond genetic predisposition. In model 6, after controlling for overall health rating, the HR was 2.76 (95% CI, 2.35-3.24). In model 7, after adjusting for symptoms of depression and anxiety, the HR for loneliness was 2.38 (95% CI, 1.88-3.00), confirming its continued significance as a predictor of SZ. Finally, in the fully adjusted model (model 8), which included socioeconomic factors, lifestyle variables, PRS, overall health status, and symptoms for depression and anxiety, the HR was 1.84 (95% CI, 1.36-2.49). This finding suggests that individuals experiencing loneliness have nearly double the risk of developing SZ. The Schoenfeld residuals test confirmed that the proportional hazards assumption was met (*P* > .05), supporting the validity of our findings. Overall, these results suggest that loneliness is independently associated with a markedly increased risk of developing SZ, and this association persists even after comprehensive adjustment for various potential confounders.

**Figure 1:**
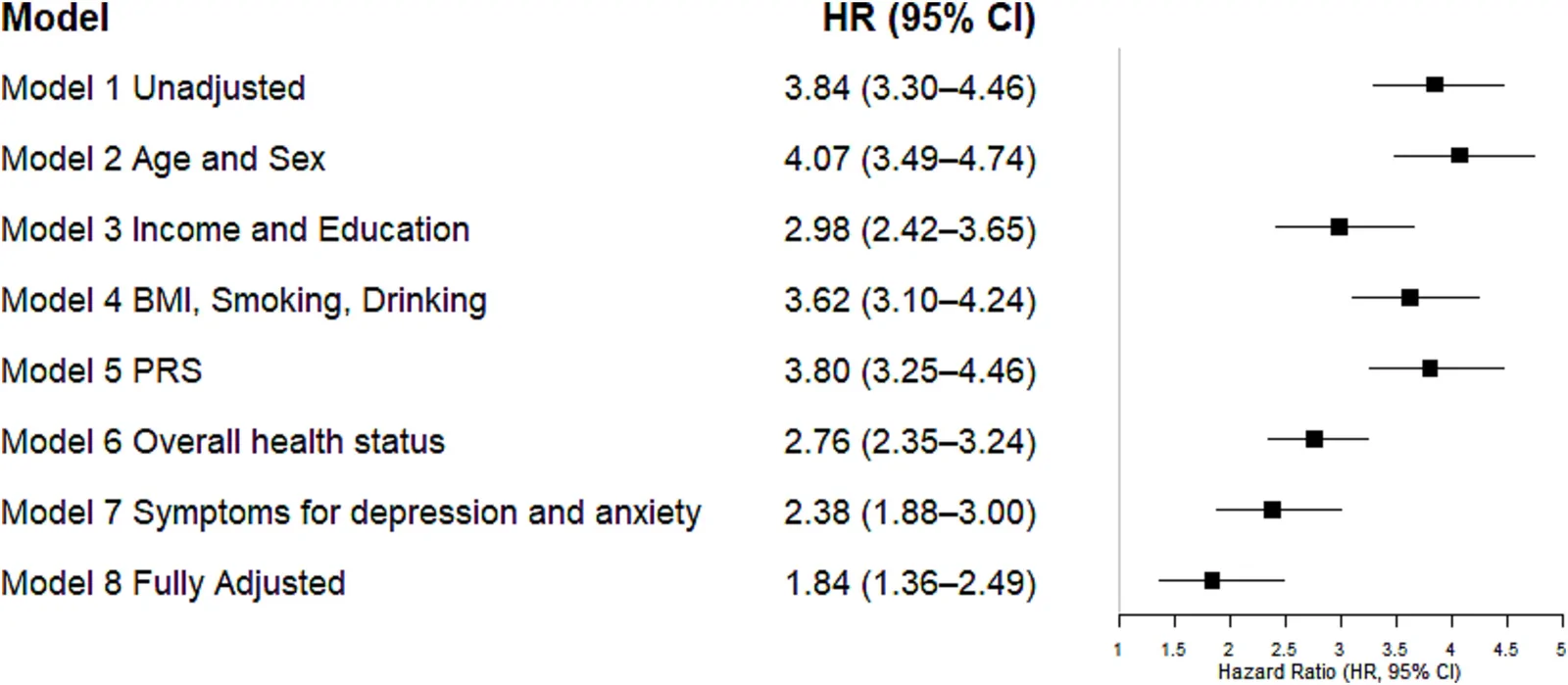
Association of loneliness with newly developed schizophrenia in models with different sets of covariates and the fully adjusted model. Each bar represents the hazard ratio for loneliness, with 95% Confidence intervals indicated. Models adjusted for various covariates, starting from age and sex in model 2 through to the fully adjusted model 8

The results of the logistic regression analysis examining the relationship between loneliness and brain volumes showed that GMV was significantly associated with loneliness (*P* = 8.46 × 10^−5^, *z* = −3.93), indicating that individuals reporting loneliness tend to have lower GMV. In contrast, WMV was not significantly related to loneliness (*P* = .118, *z* = 1.56), suggesting no meaningful association. Based on the regional analysis, 19 out of 34 cortical regions and 4 out of 8 subcortical regions showed significant FDR-corrected (*P* < .05) associations. All findings were negative, indicating that individuals reporting loneliness had smaller volumes in these regions. Key areas include middle frontal gyrus, fusiform, inferior parietal lobule, inferior temporal gyrus, lateral and medial orbitofrontal cortices, middle temporal gyrus, paracentral lobule, pericalcarine, postcentral cortex, posterior cingulate cortex, precentral gyrus, rostral anterior cingulate cortex, superior parietal lobule, superior temporal gyrus, supramarginal gyrus, temporal pole, insula, as well as nucleus accumbens, hippocampus, putamen, and thalamus. The detailed statistics for the brain regions survived FDR correction were illustrated in Table 2.

**Table 2:** Significant Logistic Regression Results for Loneliness and Brain Regions After BH-FDR Correction

Brain area	OR (CI)^a^	*Z* value	*P* value
Banks of superior temporal sulcus	0.946 (0.923-0.969)	−4.58	<.001
Caudal middle frontal gyrus	0.959 (0.937-0.982)	−3.46	<.001
Fusiform gyrus	0.961 (0.937-0.985)	−3.09	.002
Inferior parietal lobule	0.947 (0.924-0.971)	−4.28	<.001
Inferior temporal gyrus	0.956 (0.932-0.981)	−3.45	<.001
Lateral orbitofrontal cortex	0.959 (0.935-0.983)	−3.28	<.001
Medial orbitofrontal cortex	0.944 (0.920-0.968)	−4.46	<.000
Middle temporal gyrus	0.940 (0.916-0.965)	−4.70	<.001
Paracentral lobule	0.971 (0.948-0.994)	−2.46	.014
Pericalcarine cortex	0.969 (0.947-0.992)	−2.67	.007
Postcentral gyrus	0.966 (0.943-0.990)	−2.81	.005
Posterior cingulate cortex	0.969 (0.946-0.992)	−2.58	.010
Precentral gyrus	0.953 (0.930-0.977)	−3.80	<.001
Rostral anterior cingulate cortex	0.959 (0.936-0.982)	−3.41	<.001
Superior parietal lobule	0.970 (0.947-0.994)	−2.46	.014
Superior temporal gyrus	0.968 (0.944-0.992)	−2.56	.010
Supramarginal gyrus	0.951 (0.927-0.975)	−3.94	<.001
Temporal pole	0.945 (0.921-0.969)	−4.37	<.001
Insula	0.937 (0.913-0.962)	−4.92	<.001
Nucleus accumbens	0.941 (0.917-0.965)	−4.74	<.001
Hippocampus	0.965 (0.942-0.989)	−2.82	.005
Globus putamen	0.970 (0.945-0.996)	−2.25	.025
Thalamus	0.915 (0.891-0.940)	−6.59	<.001

Abbreviations: SD, standard deviation; OR, odds ratio; CI, confidence interval.

a The associations between brain regions and loneliness were assessed using the logistic regression model in 62 617 participants. The results are presented as odds ratios with 95% confidence intervals. Benjamini–Hochberg False Discovery Rate (BH-FDR) correction.

As illustrated in Figure 2, the stratified logistic regression results by genetic risk groups showed that in the low-risk group, only the supramarginal gyrus (*P* = .0412, *z* = −3.30) remained significant after FDR correction. In the middle-risk group, only the insula (*P* = .0247, *z* = −3.28) and the thalamus (*P* = .0247, *z* = −3.24) was significant. In the high-risk group, several regions showed significant volume reductions after FDR correction. These included the caudal middle frontal gyrus (*P* = .044, *z* = −2.41), entorhinal cortex (*P* = .040, *z* = −2.50), inferior temporal gyrus (*P* = .005, *z* = −3.47), lateral orbitofrontal cortex (*P* = .026, *z* = −2.74), medial orbitofrontal cortex (*P* = .005, *z* = −3.55), middle temporal gyrus (*P* = .044, *z* = −2.43), superior parietal lobule (*P* = .023, *z* = −2.84), temporal pole (*P* = .005, *z* = −3.54), insula (*P* = .039, *z* = −2.56), hippocampus (*P* = .039, *z* = −2.53), nucleus accumbens (*P* = .007, *z* = −3.36), and precentral gyrus (*P* = .018, *z* = −3.01). The thalamus demonstrated the most significant reduction with a *P*-value of 0.00027 and a *z* score of −4.51. All these significant associations were negative, indicating smaller regional volumes among individuals reporting loneliness. The detailed statistics for the brain regions survived FDR correction were reported in Table 3.

**Figure 2:**
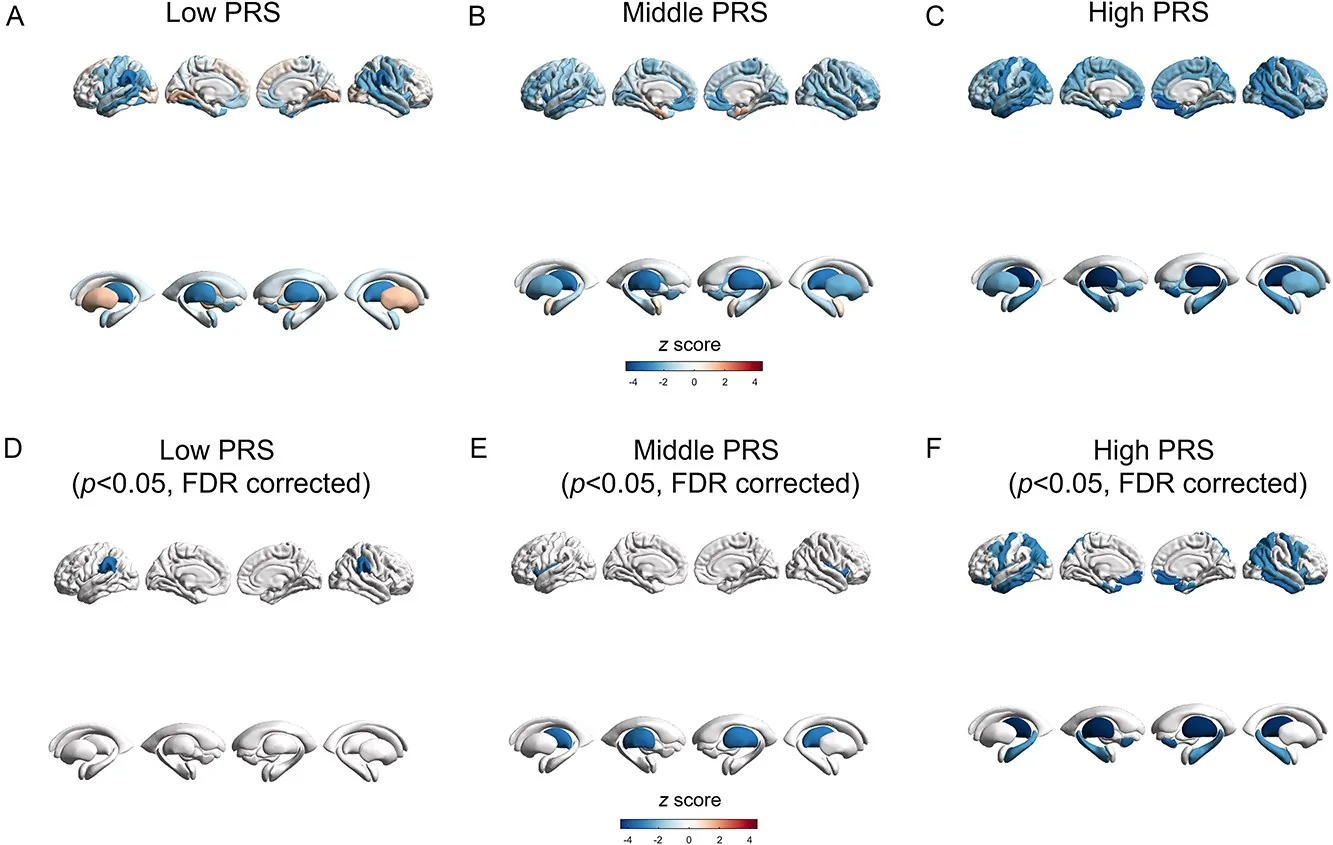
Associations between loneliness and brain imaging phenotypes. (A–C) Association between loneliness and brain imaging phenotypes stratified by genetic risk groups (Low/Middle/High). (D–F) The Same Associations That Remained Statistically Significant After Benjamini–Hochberg false discovery rate (BH-FDR) correction based on *P*-Value thresholds. The brain imaging phenotypes analyzed included cortical and subcortical volumes. Associations between loneliness and brain structures, stratified by genetic risk groups were estimated for 62 617 participants with available neuroimaging data using logistic regression models. Results are expressed as *Z* scores. A greater absolute value of the *Z* score indicates a stronger negative association between loneliness and the brain imaging phenotypes, Reflecting how far the observed values deviate from the mean. *P*-Values were adjusted using the BH-FDR method to account for multiple comparisons, with a two-sided *P* value <.05 indicating statistical significance

**Table 3:** Significant Stratified Logistic Regression Results by Genetic Risk Groups After BH-FDR Correction

Group	Brain area	OR (CI)^a^	*Z* value	*P* value
Low	Supramarginal gyrus	0.927 (0.886-0.970)	−3.30	<.001
Middle	Insula	0.927 (0.886-0.970)	−3.28	.001
Middle	Thalamus	0.926 (0.885-0.970)	−3.24	.001
High	Banks of superior temporal sulcus	0.941 (0.903-0.981)	−2.88	.004
High	Caudal middle frontal gyrus	0.951 (0.913-0.991)	−2.41	.016
High	Entorhinal cortex	0.950 (0.912-0.989)	−2.50	.013
High	Inferior parietal lobule	0.940 (0.901-0.982)	−2.81	.005
High	Inferior temporal gyrus	0.925 (0.884-0.966)	−3.47	<.001
High	Lateral orbitofrontal cortex	0.941 (0.901-0.983)	−2.74	.006
High	Medial orbitofrontal cortex	0.923 (0.884-0.965)	−3.55	<.001
High	Middle temporal gyrus	0.946 (0.905-0.989)	−2.43	.015
High	Precentral gyrus	0.936 (0.897-0.977)	−3.01	.003
High	Superior parietal lobule	0.941 (0.903-0.981)	−2.84	.005
High	Temporal pole	0.923 (0.883-0.965)	−3.54	<.001
High	Insula	0.943 (0.902-0.987)	−2.56	.0106
High	Nucleus accumbens	0.928 (0.888-0.969)	−3.36	<.001
High	Hippocampus	0.947 (0.908-0.988)	−2.53	.0113
High	Thalamus	0.900 (0.860-0.942)	−4.51	<.001

Abbreviations: SD, standard deviation; OR, odds ratio; CI, confidence interval.

a The associations between brain regions and loneliness were assessed using the logistic regression model in low, middle, and high genetic risk groups. The results are presented as odds ratios with 95% confidence intervals. Benjamini–Hochberg False Discovery Rate (BH-FDR) correction.

## Discussion

This large-scale longitudinal study provides robust evidence that loneliness is independently associated with an increased risk of developing SZ, even after adjusting for demographic, socioeconomic, lifestyle, and genetic factors. The findings underscore the importance of social experiences in the etiology of SZ and suggest that loneliness may serve as a modifiable risk factor for the disorder.

Our analysis demonstrated that individuals reporting loneliness had nearly a 2-fold higher hazard of incident SZ. This aligns with prior research indicating that social isolation and loneliness are linked to adverse mental health outcomes, including psychosis.^8^^,^^26^^,^^27^ Importantly, the association remained significant after adjusting for PRS, suggesting that loneliness contributes to SZ risk beyond genetic predisposition. This highlights the potential for social interventions to mitigate disease onset, regardless of genetic susceptibility.

Neuroimaging results further elucidate the neurobiological correlates of loneliness. Specifically, individuals reporting loneliness exhibited lower global GMV, with regional reductions observed in multiple cortical and subcortical areas, including the prefrontal cortex, temporal regions, insula, hippocampus, nucleus accumbens, and thalamus. These regions are critically involved in social cognition, emotional regulation, reward processing, and sensory integration—functions often disrupted in SZ.^28-30^ The observed volumetric reductions support the hypothesis that loneliness may influence brain structure, potentially contributing to or reflecting underlying neurobiological vulnerabilities.

Besides, while only specific regions showed significant volume reductions among low- and middle-genetic risk groups, the high genetic risk group exhibited widespread structural deficits associated with loneliness. Notably, regions such as the caudal middle frontal gyrus, entorhinal cortex, hippocampus, and thalamus demonstrated the most substantial volume reductions in high-risk individuals. These findings suggest that genetic predisposition may modulate the neurobiological impact of loneliness, with high genetic risk amplifying structural vulnerabilities. This gene–environment interaction warrants further investigation to understand the mechanisms by which social factors and genetic susceptibility jointly influence brain development and SZ risk.^31^^,^^32^

The neurobiological implications of these findings are multifaceted. Reduced volumes in regions like the hippocampus and prefrontal cortex align with existing literature linking structural abnormalities to negative symptoms and cognitive deficits in SZ.^33-35^ Specifically, prior research indicated that negative symptoms were associated with a temporal continuum of orbitofrontal abnormalities that may predate the occurrence of SZ.^36^ Additionally, a meta-analysis of 25 structural MRI studies found that structural brain abnormalities were consistently located in the prefrontal, temporal, limbic, and subcortical regions in SZ with persistent negative symptoms including blunted affect, alogia, asociality, and anhedonia.^37^ Moreover, brain structures such as the insula and hippocampus are critically involved in higher-level cognitive domains like executive function and social cognition, all of which are functions often disrupted in SZ.^38^ The involvement of limbic and reward-related areas, such as the nucleus accumbens and insula, may underlie the social anhedonia and emotional dysregulation observed in lonely individuals and those at risk for SZ.^39^^,^^40^ Additionally, the pronounced thalamic volume reduction in high-risk individuals underscores the importance of thalamocortical circuits in the pathophysiology of SZ and social cognition. The observed reductions in brain volume suggest that loneliness may affect brain structure, which could contribute to underlying neurobiological vulnerabilities linked to symptoms such as negative symptoms and cognitive deficits in SZ. This highlights the potential for interventional or mechanistic studies to explore whether reducing loneliness could lower the risk of developing SZ.

To address loneliness, interventions might involve promoting social support programs that enhance community engagement, launching public health campaigns to raise awareness about loneliness, and leveraging digital platforms to facilitate social interaction.^41^^,^^42^ Furthermore, therapeutic approaches such as cognitive-behavioral therapy can equip individuals with coping strategies, while policy initiatives can foster inclusive environments that bolster mental health resources.^42^^,^^43^ These approaches highlight the need for further research into whether reducing loneliness can effectively lower the risk of developing SZ.

### Limitations

The study also has several limitations. Firstly, as an observational study, it cannot establish cause-and-effect relationships and cannot rule out the possibility that reverse causality might partly explain the observed associations. Secondly, although the UK Biobank’s recruitment process was efficient and enabled the enrollment of around 500 000 participants, the response rate was only ~5.5%, which could lead to selection bias.^44^ Thirdly, even after adjusting for many potential confounding variables, there remains a possibility of residual confounding from unmeasured or unknown factors. Besides, the UK Biobank is a prospective cohort study of middle-aged and older population, so cautiousness is needed when generalizing our study findings to the entire population. Lastly, a single yes or no question may overlook the varying degrees of loneliness, failing to account for nuances such as situational loneliness versus chronic loneliness. This binary approach might not reflect the emotional depth or intensity of loneliness, potentially leading to an underestimation of its prevalence and impact. Such limitations could affect the reliability of our findings and their comparability with studies that utilize multi-item scales, which provide a richer, more detailed view of loneliness. Moreover, our measure captures loneliness at a single time point, preventing us from observing time-varying associations. However, previous research indicates that loneliness trajectories among older individuals are relatively stable,^45^ suggesting that a single assessment can serve as a valid indicator of long-term exposure. While these limitations may affect the comparability of our findings with other studies, prior research with multi-scale assessments has similarly found a decrease in brain volume associated with increased loneliness, supporting the consistency of our results.^46-48^

## Conclusion

In summary, our findings highlight loneliness as a significant, independent predictor of SZ risk, with associated structural brain alterations that are modulated by genetic vulnerability. These results underscore the importance of social factors in the neurobiological pathways of SZ and point toward potential avenues for early intervention. Addressing loneliness could be a promising strategy in reducing the burden of SZ, particularly in high-risk populations.

## Supplementary Material

Supplementary_Tables_sbaf179

## Data Availability

The data could be accessed from the following link: https://www.ukbiobank.ac.uk.
