# Supplementary material for: Loneliness, Genetic Susceptibility, Brain Structure, and the Risk of Schizophrenia: A Large Prospective Study
Source: Schizophr Bull. 2025 Oct 16;52(5):sbaf179. doi: 10.1093/schbul/sbaf179 (PMC13645836; doi:10.1093/schbul/sbaf179)
Supplement: Supplementary file 1 [file supplementary_tables_sbaf179.docx]

**Supplementary Table 1. Descriptive statistics for brain phenotypes.**

| **Brain structure** | **Mean (SD)** | **25th** | **50th** | **75th** |
| --- | --- | --- | --- | --- |
| **Volume of subcortical structures (mm^3^)** | | | | |
| Nucleus Accumbens | 430.02 (105.05) | 358.0 | 429.0 | 499.5 |
| Amygdala | 1238.82 (217.69) | 1090.5 | 1234.0 | 1380.0 |
| Caudate nucleus | 3447.48 (421.38) | 3158.0 | 3425.0 | 3711.0 |
| Hippocampus | 3794.54 (445.00) | 3508.5 | 3794.5 | 4079.0 |
| Globus pallidus | 1764.08 (233.22) | 1609.5 | 1746.0 | 1897.5 |
| Putamen | 4743.48 (580.42) | 4348.0 | 4718.5 | 5115.0 |
| Thalamus | 7582.87 (740.89) | 7073.5 | 7551.5 | 8055.0 |
| Globus pallidus | 1761.98 (234.44) | 1607.0 | 1744.0 | 1896.0 |
| **Volume of cortical structures (mm^3^)** | | | | |
| Caudal anterior cingulate cortex | 1858.65 (398.11) | 1591.0 | 1841.5 | 2102.5 |
| Caudal middle frontal gyrus | 6469.20 (981.34) | 5785.0 | 6413.5 | 7086.5 |
| Frontal pole | 1069.95 (134.02) | 978.5 | 1062.0 | 1152.5 |
| Lateral orbitofrontal cortex | 7988.24 (851.62) | 7392.5 | 7953.0 | 8544.0 |
| Medial orbitofrontal cortex | 5948.88 (648.08) | 5496.5 | 5913.5 | 6367.5 |
| Paracentral lobule | 3985.62 (497.44) | 3641.0 | 3956.5 | 4298.0 |
| Pars opercularis | 4463.57 (617.60) | 4030.0 | 4412.0 | 4839.0 |
| Pars orbitalis | 2655.71 (329.93) | 2425.5 | 2639.5 | 2866.5 |
| Pars triangularis | 4061.65 (571.52) | 3658.5 | 4016.0 | 4423.5 |
| Precentral gyrus | 13976.94 (1599.97) | 12878.5 | 13915.0 | 15014.5 |
| Rostral anterior cingulate cortex | 2504.63 (432.20) | 2205.5 | 2477.0 | 2779.0 |
| Rostral middle frontal gyrus | 16024.97 (2158.99) | 14504.5 | 15864.0 | 17396.5 |
| Superior frontal gyrus | 22984.46 (2734.85) | 21051.5 | 22809.0 | 24704.0 |
| Banks of superior temporal sulcus | 2401.79 (347.04) | 2161.5 | 2376.5 | 2617.0 |
| Entorhinal cortex | 1889.07 (334.27) | 1659.5 | 1862.0 | 2090.0 |
| Fusiform gyrus | 10153.02 (1232.93) | 9293.5 | 10088.0 | 10947.0 |
| Inferior temporal gyrus | 11554.91 (1520.57) | 10484.5 | 11464.5 | 12527.5 |
| Middle temporal gyrus | 11756.57 (1512.30) | 10694.5 | 11672.5 | 12737.0 |
| Parahippocampal gyrus | 1968.82 (256.09) | 1794.0 | 1956.5 | 2130.0 |
| Superior temporal gyrus | 12436.46 (1460.86) | 11413.0 | 12357.5 | 13370.5 |
| Transverse temporal gyrus | 1117.04 (180.65) | 989.5 | 1102.5 | 1229.0 |
| Temporal pole | 9302.19 (1171.75) | 8490.0 | 9239.0 | 10052.0 |
| Inferior parietal lobule | 13671.57 (1846.33) | 12378.0 | 13557.0 | 14857.0 |
| Isthmus cingulate | 2660.12 (387.90) | 2388.0 | 2630.5 | 2901.5 |
| Postcentral gyrus | 9993.13 (1251.18) | 9119.0 | 9923.5 | 10790.5 |
| Posterior cingulate cortex | 3363.38 (465.75) | 3049.0 | 3342.5 | 3659.0 |
| Precuneus | 10446.65 (1290.86) | 9543.0 | 10365.5 | 11272.0 |
| Superior parietal lobule | 13871.98 (1703.83) | 12685.5 | 13792.5 | 14978.5 |
| Supramarginal gyrus | 11354.83 (1573.90) | 10245.0 | 11247.0 | 12363.0 |
| Cuneus | 3171.46 (526.66) | 2798.0 | 3121.0 | 3491.5 |
| Lateral occipital cortex | 12575.56 (1677.62) | 11390.5 | 12468.0 | 13655 |
| Lingual gyrus | 6594.18 (1057.37) | 5860.5 | 6547.5 | 7279.5 |
| Pericalcarine cortex | 2246.23 (434.72) | 1935.5 | 2201.5 | 2513.5 |
| Insula | 7315.91 (795.67) | 6750.5 | 7262.0 | 7829.0 |

Note: SD, standard deviation.

**Supplementary Table 2. Supplementary Descriptive Statistics for the Overall Sample and by Baseline Loneliness and Incident schizophrenia**

| Characteristics | Participants ^a^ | | | | |
| --- | --- | --- | --- | --- | --- |
|  | Total  (N=491,163) | Loneliness at baseline | | Incident schizophrenia | |
|  |  | No (n=400,541) | Yes (n=91,072) | No  (n=490938) | Yes (n=675) |
| Ethnicity | | | | | |
| White | 446170 (90.8) | 365426 (91.2) | 80744 (88.7) | 445605 (90.8) | 565 (83.7) |
| Multiracial | 18308 (3.7) | 14070 (3.5) | 4238 (4.7) | 18254 (3.7) | 54 (8.0) |
| Asian | 17010 (3.5) | 13427 (3.4) | 3583 (3.9) | 16978 (3.5) | 32 (4.7) |
| Black | 2717 (0.6) | 2049 (0.5) | 668 (0.7) | 2711 (0.6) | 6 (0.9) |
| Chinese | 1488 (0.3) | 1236 (0.3) | 252 (0.3) | 1486 (0.3) | 2 (0.3) |
| Other | 4311 (0.9) | 3083 (0.8) | 1228 (1.3) | 4299 (0.9) | 12 (1.8) |
| Education level | | | | | |
| Degree level or professional qualification | 229725 (46.8) | 193467 (48.3) | 36258  (39.8) | 229515 (46.8) | 210 (31.1) |
| Other levels | 170158 (34.6) | 136502 (34.1) | 33656 (37.0) | 169927 (34.6) | 231 (34.2) |
| Average total household income before tax | | | | | |
| Less than £31,000 | 200936 (40.9) | 154185 (38.5) | 46751 (51.3) | 200498 (40.8) | 438 (64.9) |
| More than £31,000 | 217383 (44.3) | 187609 (46.8) | 29774 (32.7) | 217330 (44.3) | 53 (7.9) |
| BMI, mean (SD), kg m^−2^ | 27.43 (4.80) | 27.29 (4.64) | 28.06 (5.40) | 27.43 (4.79) | 29.01 (6.14) |
| Smoking status | | | | | |
| Never Smoked | 196833 (40.1) | 162228 (40.5) | 34605 (38.0) | 196626 (40.0) | 207 (30.7) |
| Ever smoked | 293054 (59.7) | 236992 (59.2) | 56062 (61.6) | 292591 (59.6) | 463 (68.6) |
| Drinking status | | | | | |
| Never | 21536 (4.4) | 16486 (4.1) | 5050 (5.5) | 21465 (4.4) | 71 (10.5) |
| Previous | 17611 (3.6) | 12768 (3.2) | 4843 (5.3) | 17521 (3.6) | 90 (13.3) |
| Current | 451955 (92.0) | 370962 (92.6) | 80993 (88.9) | 451445 (92.0) | 510 (75.6) |
| Schizophrenia polygenic risk score, mean (SD) | -0.31 (1.01) | -0.32 (1.00) | -0.24 (1.01) | -0.31 (1.00) | 0.35 (1.06) |
| Symptoms for depression and anxiety | 5.19 (1.47) | 4.96 (1.26) | 6.47 (1.83) | 5.19 (1.46) | 6.07 (1.92) |

Abbreviations: SD, standard deviation; BMI, body mass index (calculated as weight in kilograms divided by height in meters squared);

^a^ Data are presented as number (percentage) of participants unless otherwise indicated.
